# Supplementary material for: Native structure of mosquito salivary protein uncovers domains relevant to pathogen transmission
Source: Nat Commun. 2023 Feb 17;14:899. doi: 10.1038/s41467-023-36577-y (PMC9935623; doi:10.1038/s41467-023-36577-y)
Supplement: Supplementary file 1 — Supplementary Information [file 41467_2023_36577_MOESM1_ESM.pdf]

Supplementary information for

Native structure of mosquito salivary protein uncovers domains relevant to pathogen transmission

Authors: Shiheng Liu[1,2], Xian Xia[1,2], Eric Calvo[3] & Z. Hong Zhou[1,2✉]

Affiliations:

[1] Department of Microbiology, Immunology, and Molecular Genetics, University of California, Los Angeles, CA 90095, USA.

[2] California NanoSystems Institute, University of California, Los Angeles, CA 90095, USA.

[3] Laboratory of Malaria and Vector Research, National Institute of Allergy and Infectious Diseases, National Institutes of Health, Rockville, MD 20852, USA.

These authors contributed equally: Shiheng Liu, Xian Xia.

✉Corresponding author. Email: Hong.Zhou@UCLA.edu

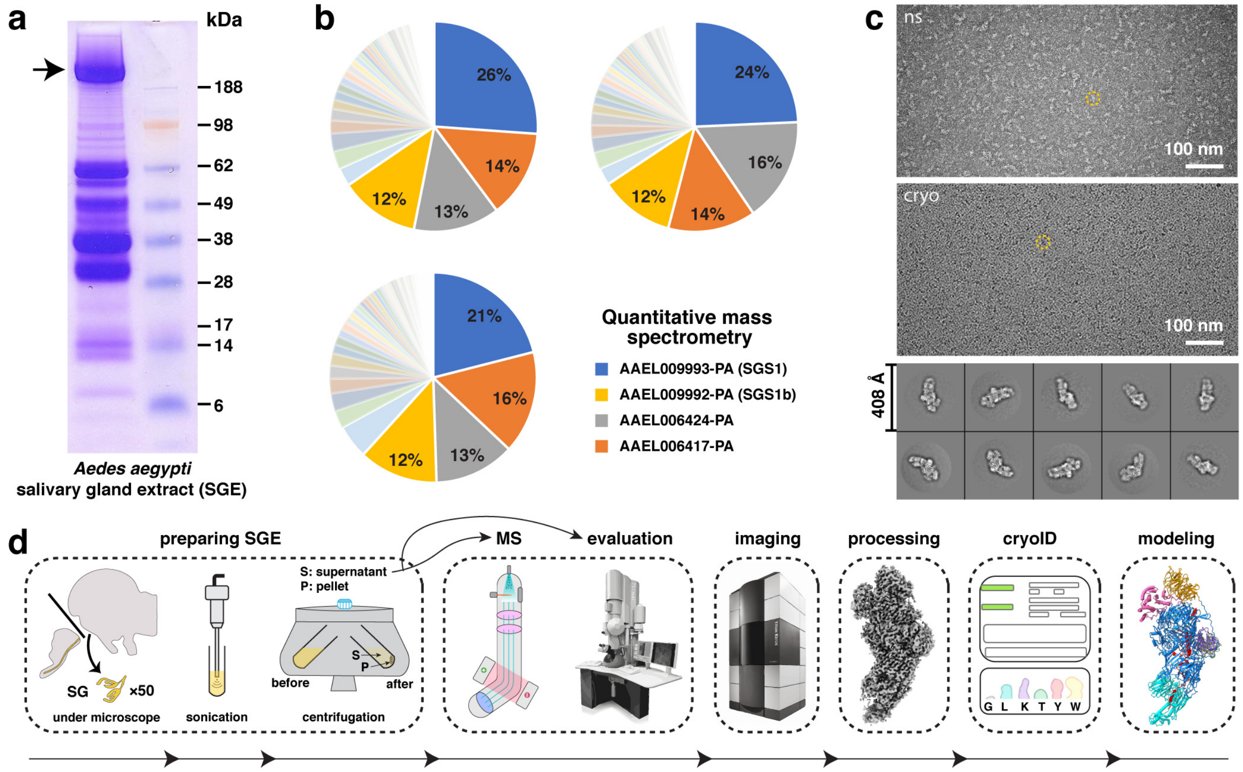

Supplementary Figure 1. Endogenous structural proteomics workflow for the salivary gland extract (SGE) of *Aedes aegypti*. a Coomassie-stained SDS-PAGE of the *Aedes aegypti* SGE. Three repeats with independent SGE samples showed reproducible result. Arrowhead denotes the band consistent with the molecular weights of the SGS1 protein. **b** Quantitative mass spectrometry showing the abundance of the proteins in the SGE of *Aedes aegypti*. **c** Representative negative stain (upper panel; from 108 micrographs) and drift-corrected cryo-EM (middle panel; from 2,408 micrographs) micrographs as well as images of 2D classification from cryo-EM (lower panel) of the SGE. Representative particles are shown in yellow dotted circles. **d** Depiction of the workflow. 50 pairs of salivary glands were dissected under a stereomicroscope and transferred into PBS buffer. SGE was obtained by disrupting the gland walls by sonication and cleared by centrifugation. The SGE was checked by SDS-PAGE, followed by mass spectrometry and negative stain EM evaluation. Cryo-EM imaging and analysis of the SGE yielded a 3.3 Å resolution cryo-EM density map. The protein in the cryo-EM map was identified using *cryoID* and then modeled *de novo*, yielding the final atomic resolution structure of the SGS1 protein.

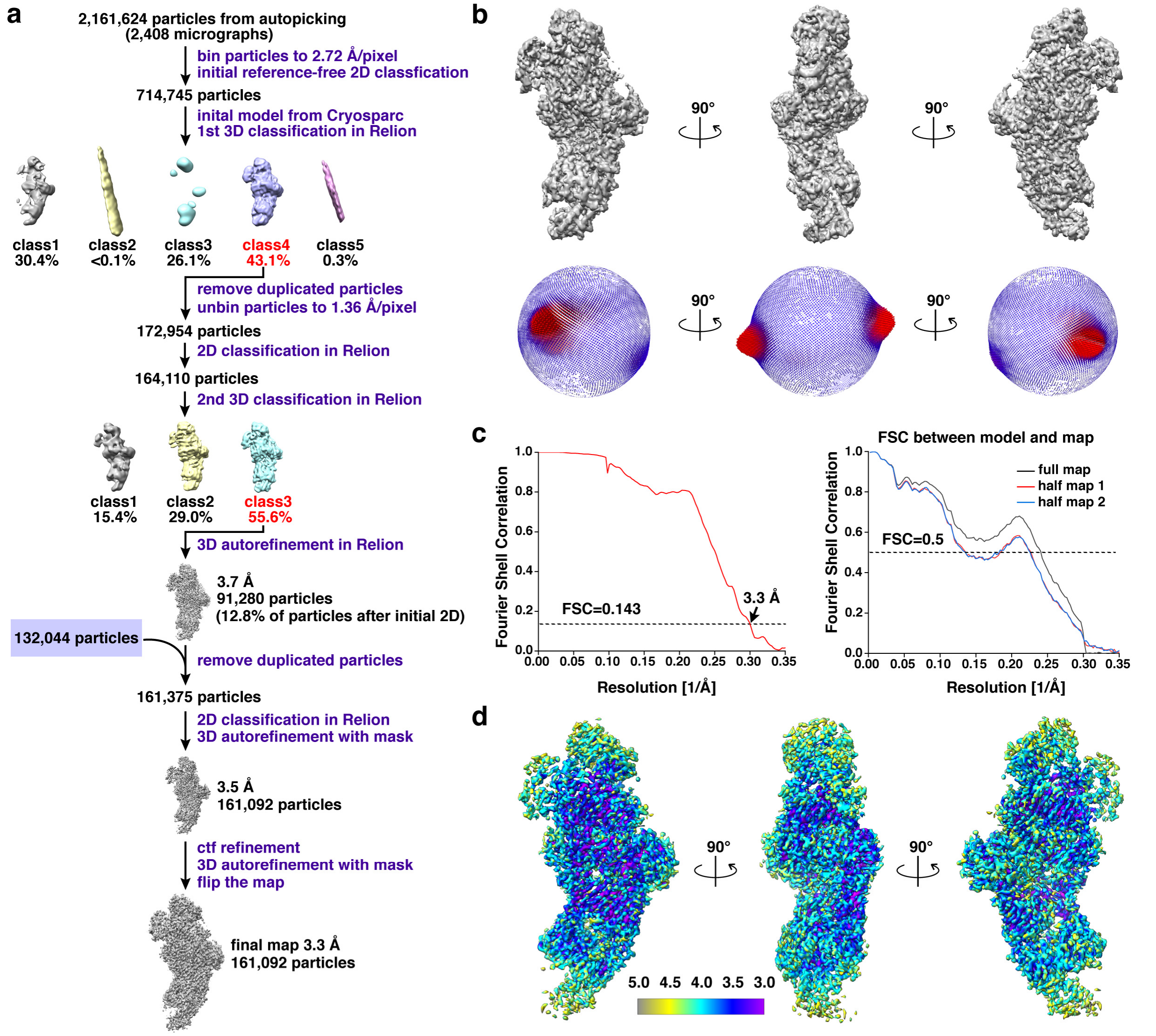

Supplementary Figure 2. The cryo-EM structure determination process for SGS1. **a** Data processing workflow. **b** Angular distribution of all particles used for the final 3.3 Å map of the SGS1 protein. **c** Left panel: FSC as a function of spatial frequency demonstrating the resolution of the final reconstruction of the SGS1 protein; right panel: FSC coefficients as a function of spatial frequency between model and cryo-EM density maps. The generally similar appearances between the FSC curves obtained with half maps with (red) and without (blue) model refinement indicate that the refinement of the atomic coordinates did not suffer from severe over-fitting. **d** Resmap local resolution estimation.

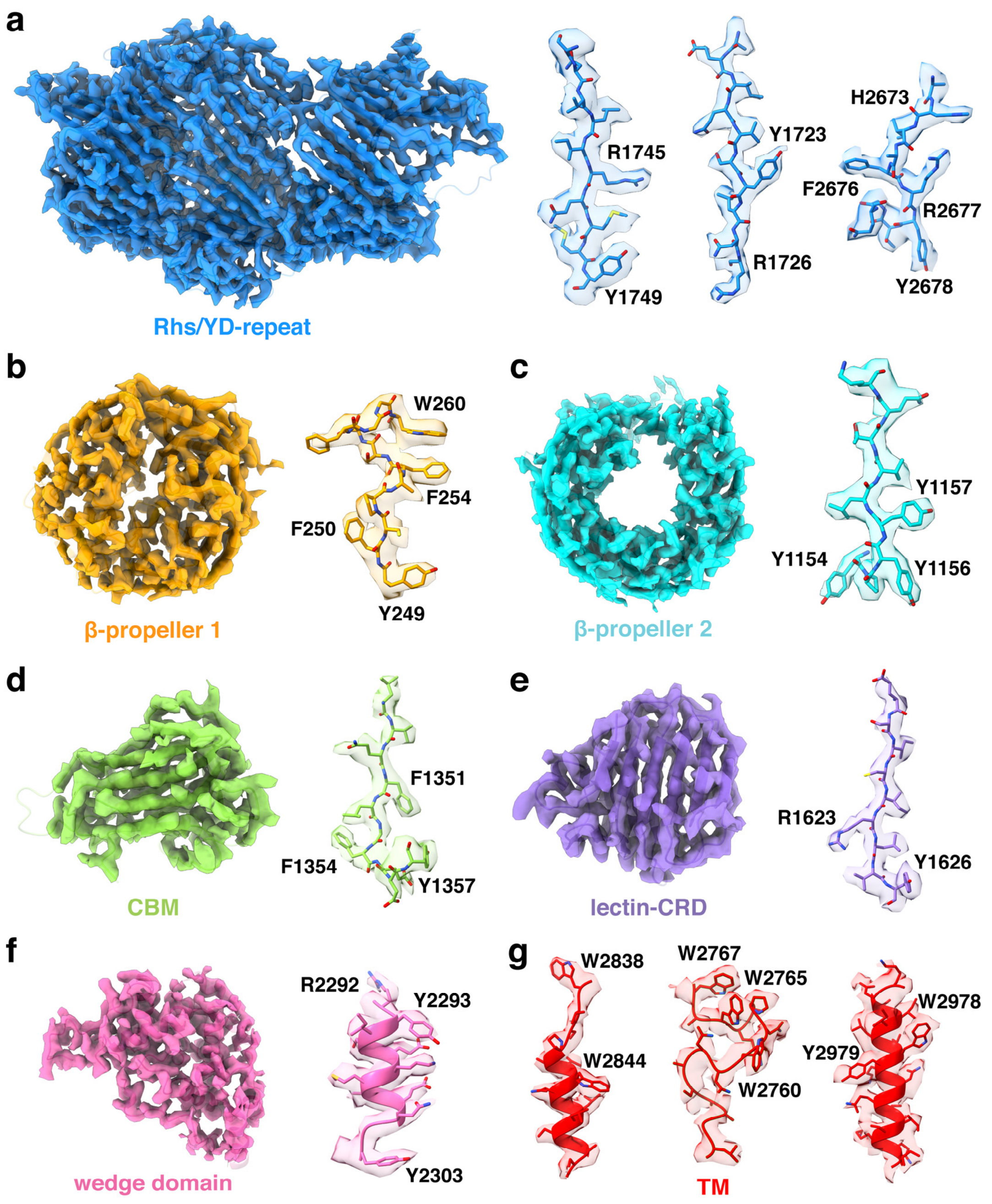

Supplementary Figure 3. Representative cryo-EM density maps of SGS1. a-g Cryo-EM density map fitted with model

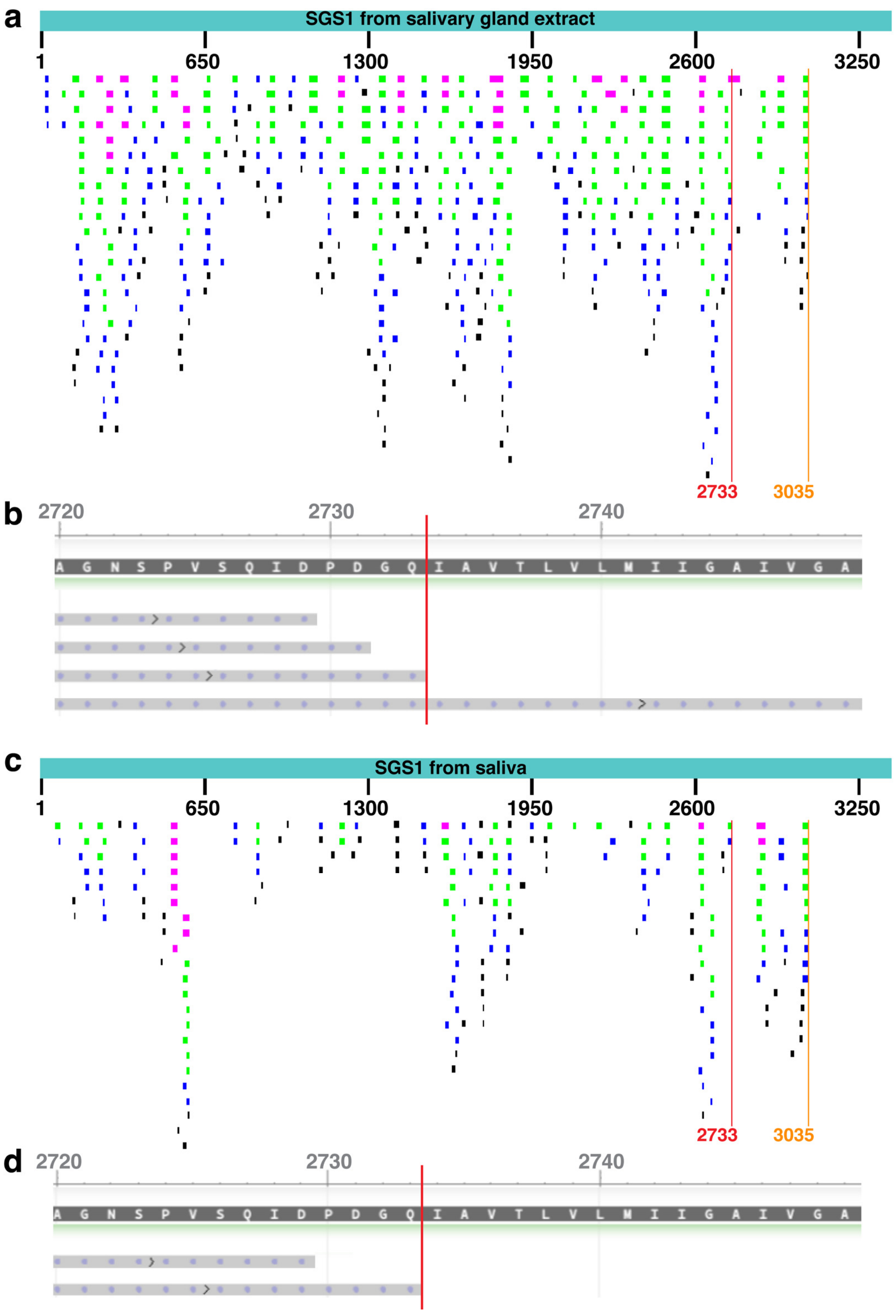

Supplementary Figure 4. Mass Spec sequence coverage of SGS1 from the salivary gland extract (a,b) and saliva (c,d) of *Aedes aegypti*. AP cleavage site of SGS1 was indicated by red solid lines. No fragments were detected after residue 3035 of SGS1 (orange solid line).

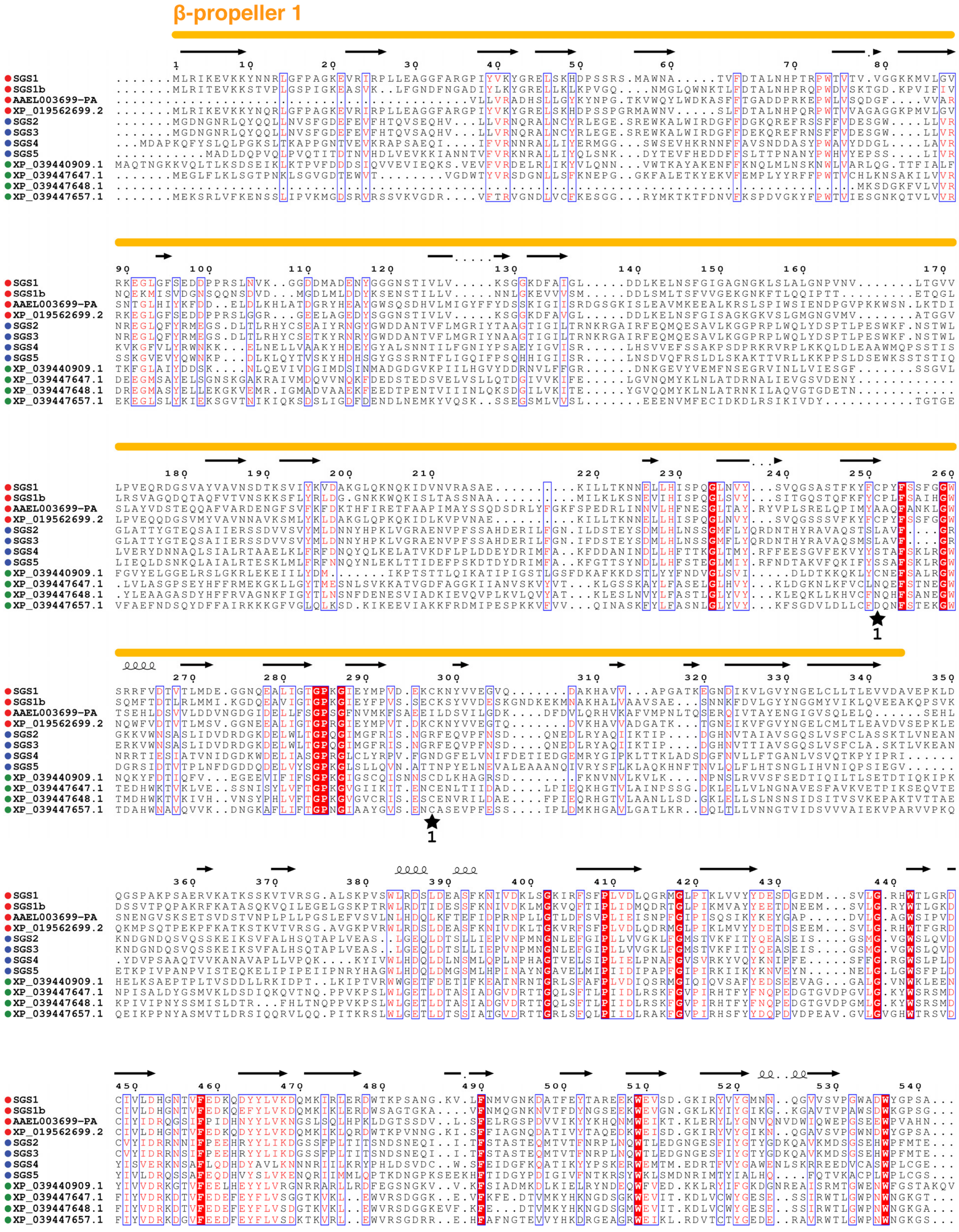
β-propeller 1
SGS1
SGS1b
AAEL003699-PA
XP_019562699.2
SGS2
SGS3
SGS4
SGS5
XP_039440909.1
XP_039447647.1
XP_039447648.1
XP_039447657.1
1
1

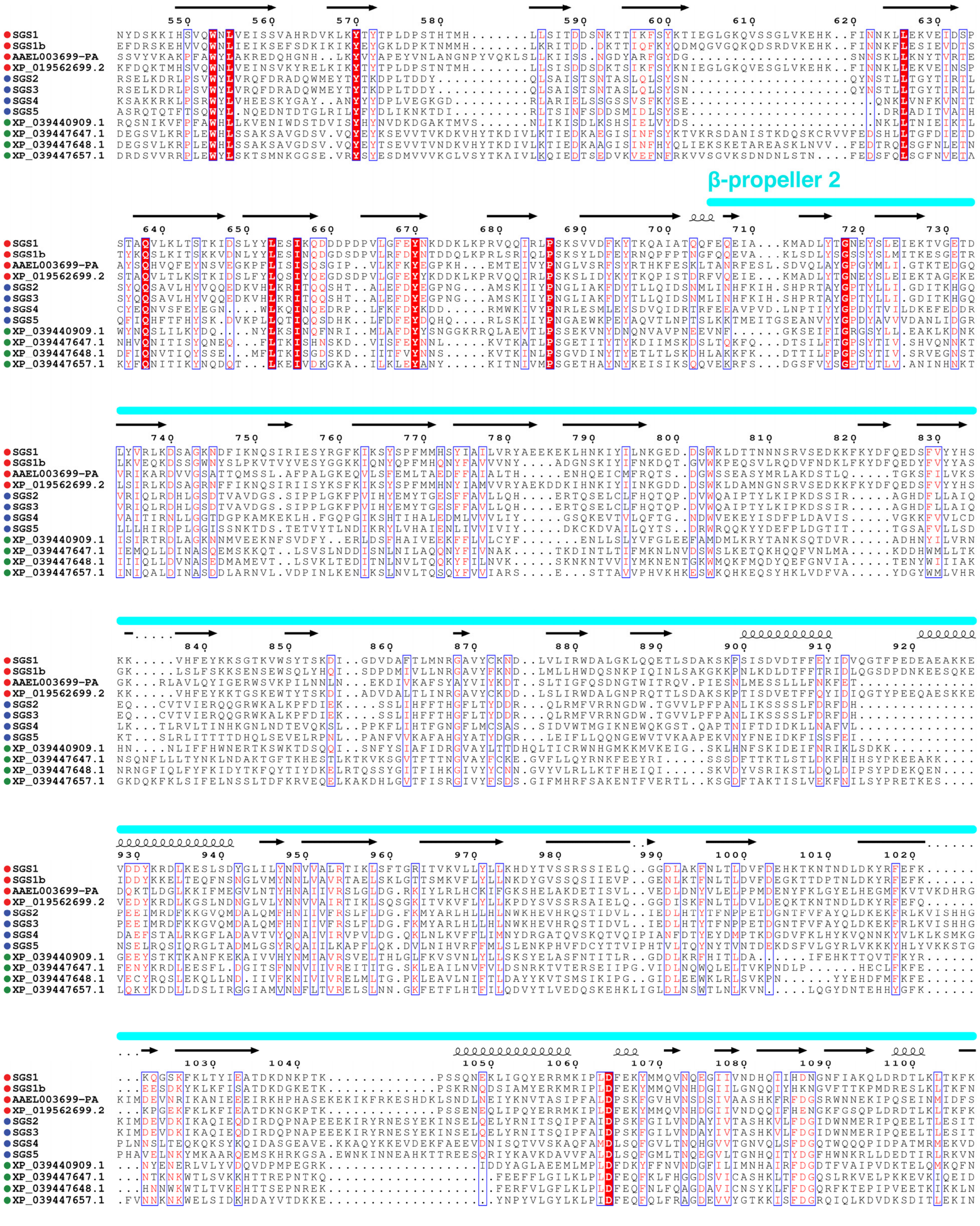
β-propeller 2

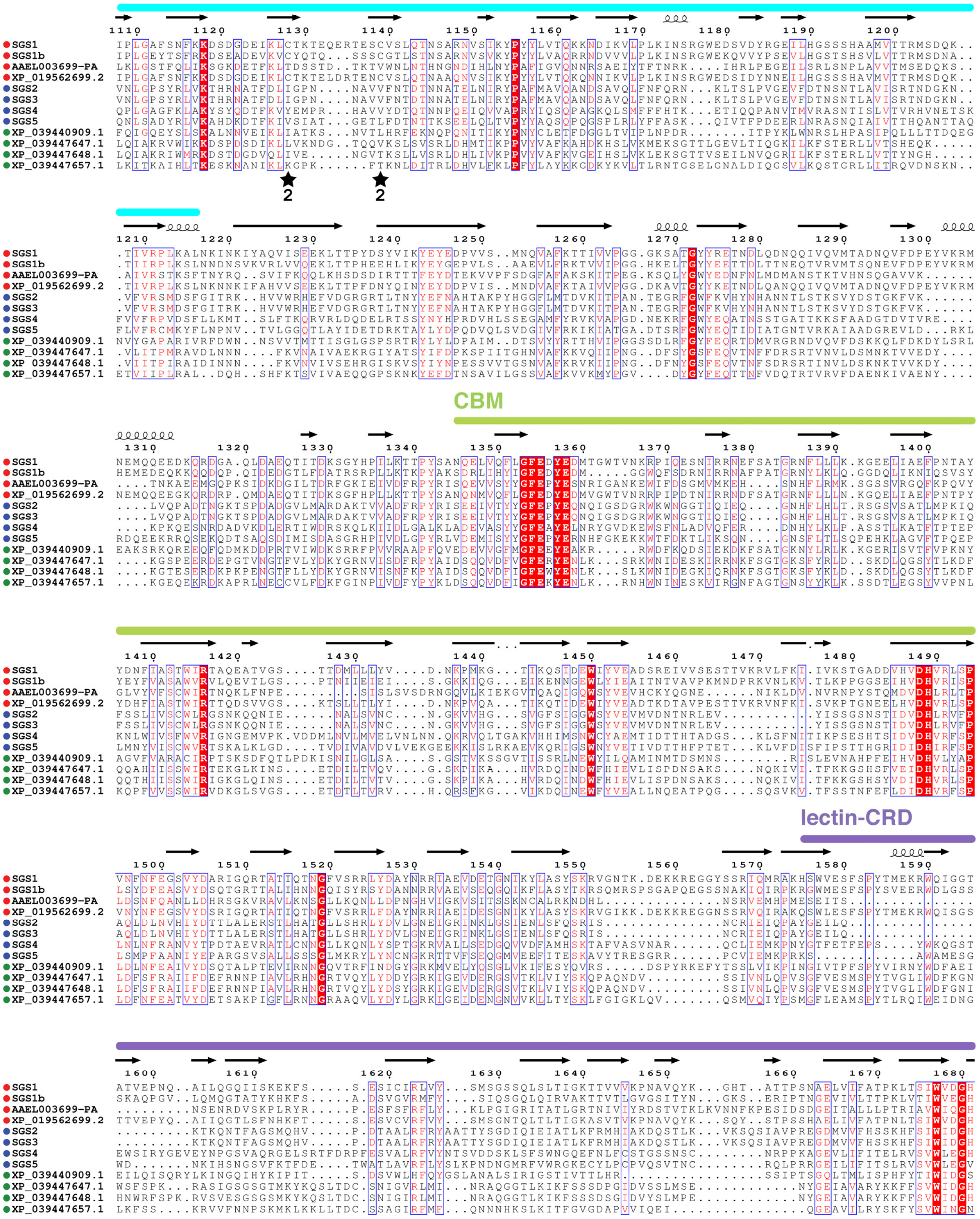
CBM
lectin-CRD
SGS1
SGS1b
AAEL003699-PA
XP_019562699.2
SGS2
SGS3
SGS4
SGS5
XP_039440909.1
XP_039447647.1
XP_039447648.1
XP_039447657.1

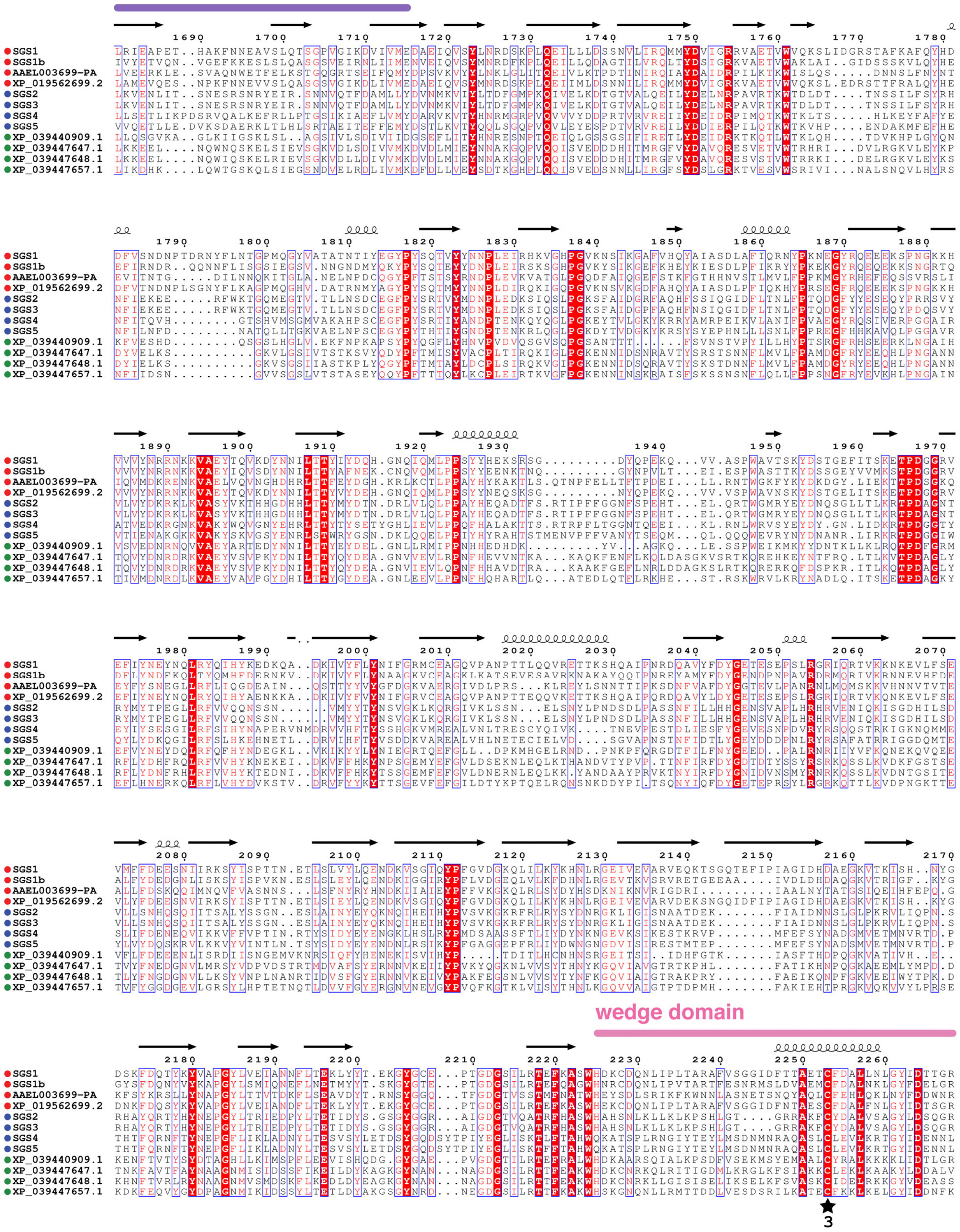
SGS1
SGS1b
AAEL003699-PA
XP_019562699.2
SGS2
SGS3
SGS4
SGS5
XP_039440909.1
XP_039447647.1
XP_039447648.1
XP_039447657.1
wedge domain
3

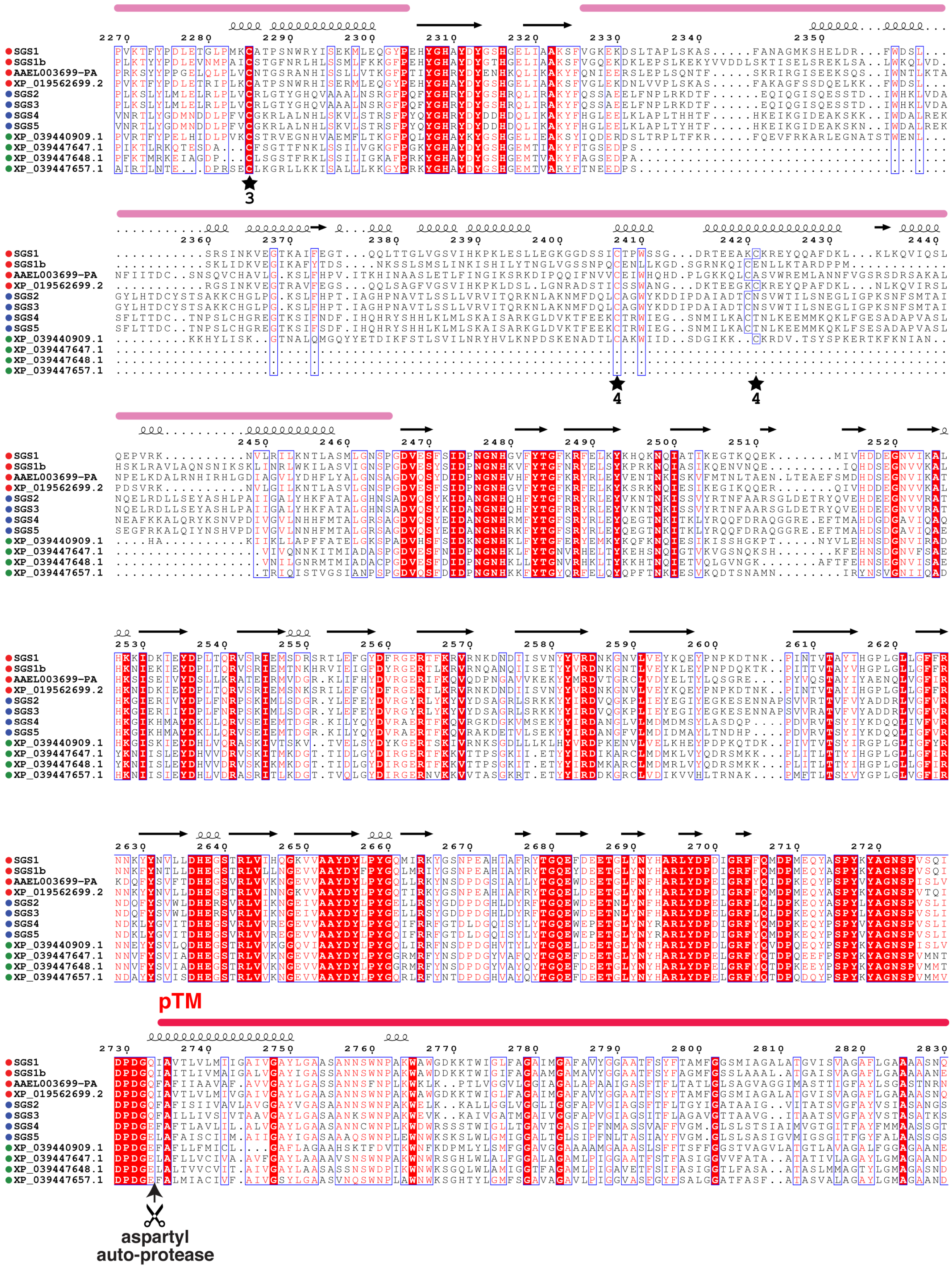
SGS1
SGS1b
AAEL003699-PA
XP_019562699.2
SGS2
SGS3
SGS4
SGS5
XP_039440909.1
XP_039447647.1
XP_039447648.1
XP_039447657.1
3
4
4
pTM
aspartyl
auto-protease

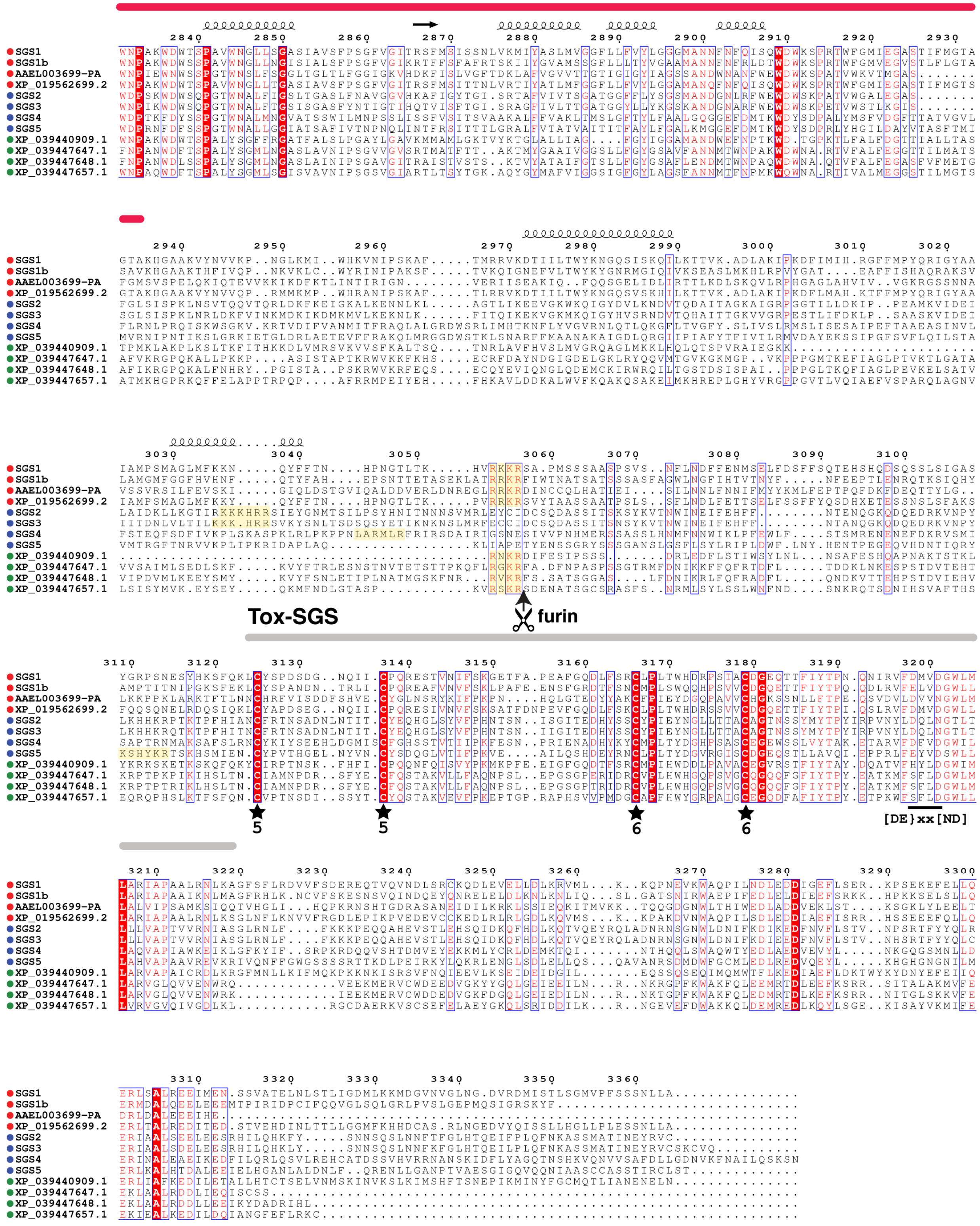

Supplementary Figure 5. Sequence alignment of the SGS proteins from different mosquito species. The 12 selected sequences are from *Aedes* (red), *Anopheles* (blue) and *Culex* (green circles). Domain information and secondary structural information are annotated on top. Protease cleavage sites (line drawing scissors) and disulfide bonds (stars) are shown on the bottom.

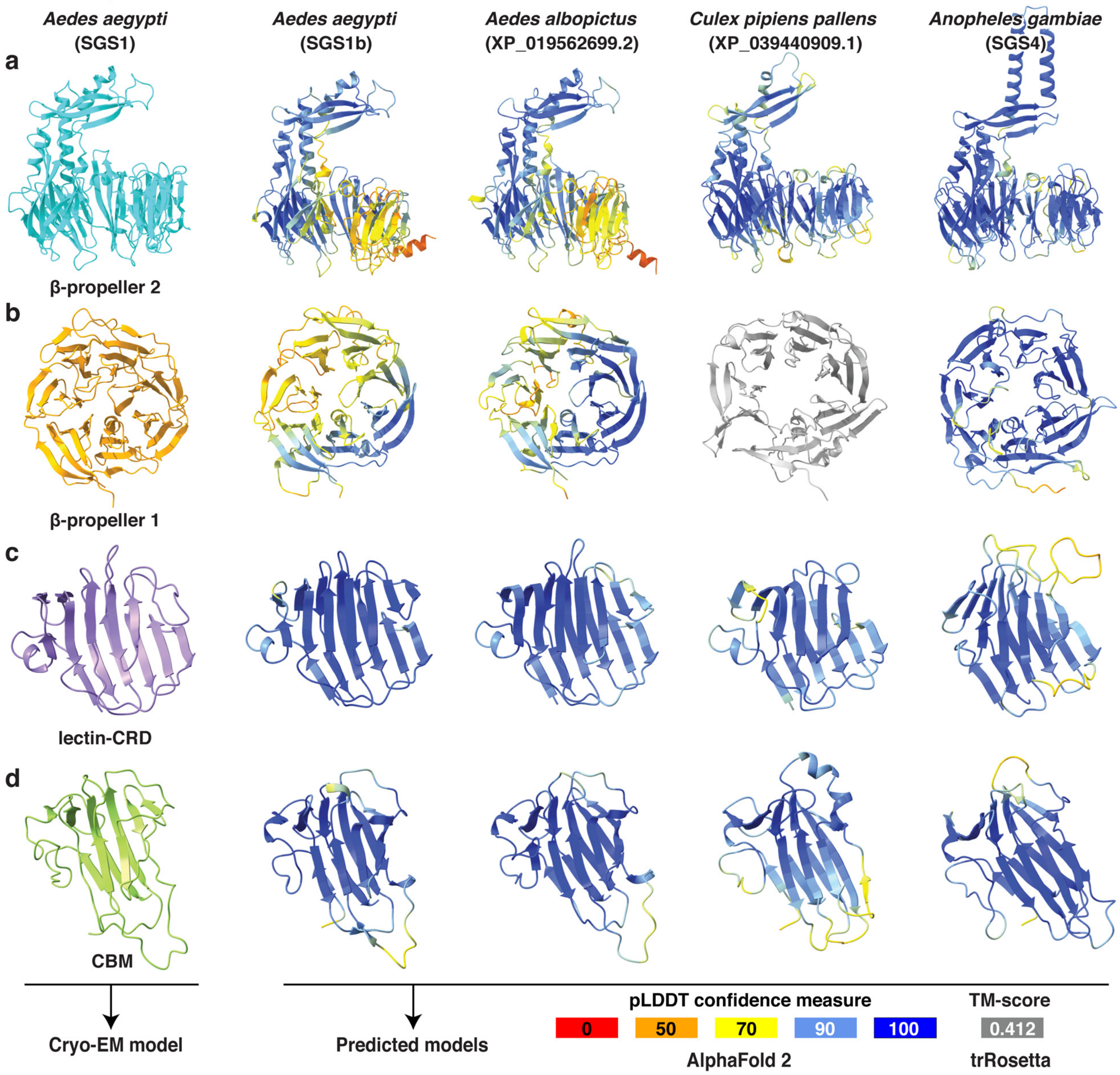

Supplementary Figure 6. Structural prediction of the SGS1 receptor domains using artificial intelligence (AI) programs. Panels from left to right: cryo-EM model of SGS1 (panel a) and the predicted models of other mosquito SGS proteins (panels b-d). All predicted models w ere generated using AlphaFold2 except for the β-propeller 1 from *Culex pipiens pallens* which w as obtained using trRosetta. For AlphaFold2 models, regions with pLDDT > 90 are expected to b e modeled to high accuracy; regions with pLDDT between 70 and 90 are expected to be modeled well; regions with pLDDT between 50 and 70 are low confidence and should be treated with caution. For the trRosetta model, TM-score higher than 0.5 usually indicates a model with correctly predicted topology.

Supplementary Table 1. Data collection and model refinement statistics

	Salivary gland surface protein 1 (SGS1) (EMD-29245) (PDB 8FJP)
**Data collection and image processing**	
Microscope	Titan Krios
Voltage (kV)	300
Camera	K2
Data collection software	SerialEM
Magnification	105,000
Pixel size at detector (Å/pixel)	1.36
Electron exposure (e–/Å^2/movie)	30
Exposure time (s)	8
Frame number	40
Defocus range (μm)	-1.5 to -3.0
Micrographs collected (no.)	2,408
Symmetry imposed	C1
Total extracted particles (no.)	2,161,624
Final particles (no.)	161,092
Map resolution (Å)	3.3
FSC threshold	0.143
Map resolution range (Å)	3.0-4.5
**Model Refinement and validation**	
Refinement software	Phenix (real space refinement)
Model resolution (Å)	4.2
FSC threshold	0.5
Map sharpening *B* factor (Å^2)	-109.4
Model composition	
Protein residues	2,932
Ligands	2
B factors (Å^2)	
Protein	50.97
Ligand	70.82
R.m.s. deviations	
Bond lengths (Å) (#>4σ)	0.005
Bond angles (°) (#>4σ)	1.177
Validation	
MolProbity score	1.72
Clashscore	6.05
Poor rotamers (%)	0.67
Ramachandran plot	
Favored (%)	94.32
Allowed (%)	5.68
Disallowed (%)	0.00
