# Supplementary material for: Native structure of mosquito salivary protein uncovers domains relevant to pathogen transmission
Source: Nat Commun. 2023 Feb 17;14:899. doi: 10.1038/s41467-023-36577-y (PMC9935623; doi:10.1038/s41467-023-36577-y)
Supplement: Supplementary file 3 — Description of Additional Supplementary Files [file 41467_2023_36577_MOESM3_ESM.pdf]

File Name: Supplementary Data 1
Description: The LC/MS results for SGE

File Name: Supplementary Data 2
Description: The LC/MS results for saliva

File Name: Supplementary Movie 1
Description: Overall structure of native SGS1 from the salivary gland extract of Aedes aegypti.

File Name: Supplementary Movie 2
Description: Architectures of Rhs/YD shell, daisy-chained helices predicted to form transmembrane helices, and receptor domains.
