# Supplementary material for: Native structure of mosquito salivary protein uncovers domains relevant to pathogen transmission
Source: Nat Commun. 2023 Feb 17;14:899. doi: 10.1038/s41467-023-36577-y (PMC9935623; doi:10.1038/s41467-023-36577-y)
Supplement: Supplementary file 8 — Reporting Summary [file 41467_2023_36577_MOESM8_ESM.pdf]

Corresponding author(s): Z. Hong Zhou
Last updated by author(s): Feb 1, 2023

Reporting Summary

Nature Portfolio wishes to improve the reproducibility of the work that we publish. This form provides structure for consistency and transparency in reporting. For further information on Nature Portfolio policies, see our Editorial Policies and the Editorial Policy Checklist.

Statistics

For all statistical analyses, confirm that the following items are present in the figure legend, table legend, main text, or Methods section.

n/a	Confirmed	
☐	☒	The exact sample size (*n*) for each experimental group/condition, given as a discrete number and unit of measurement
☐	☒	A statement on whether measurements were taken from distinct samples or whether the same sample was measured repeatedly
☒	☐	The statistical test(s) used AND whether they are one- or two-sided<br>*Only common tests should be described solely by name; describe more complex techniques in the Methods section.*
☒	☐	A description of all covariates tested
☒	☐	A description of any assumptions or corrections, such as tests of normality and adjustment for multiple comparisons
☒	☐	A full description of the statistical parameters including central tendency (e.g. means) or other basic estimates (e.g. regression coefficient) AND variation (e.g. standard deviation) or associated estimates of uncertainty (e.g. confidence intervals)
☒	☐	For null hypothesis testing, the test statistic (e.g. *F*, *t*, *r*) with confidence intervals, effect sizes, degrees of freedom and *P* value noted<br>*Give P values as exact values whenever suitable.*
☒	☐	For Bayesian analysis, information on the choice of priors and Markov chain Monte Carlo settings
☒	☐	For hierarchical and complex designs, identification of the appropriate level for tests and full reporting of outcomes
☒	☐	Estimates of effect sizes (e.g. Cohen's *d*, Pearson's *r*), indicating how they were calculated

Our web collection on statistics for biologists contains articles on many of the points above.

Software and code

Policy information about availability of computer code

Data collection	Cryo-EM: SerialEM3.7
Data analysis	Cryo-EM: MotionCor2, CTFFIND4, Relion3.0, Cryosparc v2, ResMap v1.95, cryoID, PSIPRED 4.0, AlphaFold2, Coot0.9.8.1, Phenix1.20.1, ChimeraX1.4, Chimera1.16<br>LC-MS: PEAKS v10<br>phylogenetic analysis: blastp, MUSCLE, IQ-TREE, iTOL v6<br>Multiple sequence alignment: MUSCLE, ESPript 3.0

For manuscripts utilizing custom algorithms or software that are central to the research but not yet described in published literature, software must be made available to editors and reviewers. We strongly encourage code deposition in a community repository (e.g. GitHub). See the Nature Portfolio guidelines for submitting code & software for further information.

Data

Policy information about availability of data

All manuscripts must include a data availability statement. This statement should provide the following information, where applicable:

- Accession codes, unique identifiers, or web links for publicly available datasets
- A description of any restrictions on data availability
- For clinical datasets or third party data, please ensure that the statement adheres to our policy

Cryo-EM density maps have been deposited in the Electron Microscopy Data Bank under accession numbers EMD-29245 [https://www.ebi.ac.uk/pdbe/entry/emdb/EMD-29245] (mosquito salivary gland surface protein 1). Model coordinates have been deposited in the Protein Data Bank under accession numbers 8FJP [https://doi.org/10.2210/pdb8FJP/pdb] (mosquito salivary gland surface protein 1). Other structures used in this study were obtained from the PDB with accession codes 6H6G [https://doi.org/10.2210/pdb6H6G/pdb] (TcB-TcC of Tc-toxin), 6FB3 [https://doi.org/10.2210/pdb6FB3/pdb] (Teneurin 2), 6SKA [https://doi.org/10.2210/pdb6SKA/pdb] (Teneurin 2 in complex with Latrophilin 1 Lec-Olf domains), 4NOX [http://doi.org/10.2210/pdb4NOX/pdb] (nine-bladed beta-propeller of eIF3b), 2XOM [https://doi.org/10.2210/pdb2XOM/pdb] (TmCBM61 in complex with beta-1,4- galactotriose), 3A4U [https://doi.org/10.2210/pdb3A4U/pdb] (MCFD2 in complex with carbohydrate recognition domain of ERGIC-53). Protein sequences used in this study were retrieved from Uniprot with accession ID Q16U82 [https://www.uniprot.org/uniprotkb/Q16U82/entry] (name AAEL009993-PA) and Q16U81 [https://www.uniprot.org/uniprotkb/Q16U81/entry] (name AAEL009992-PA). All other data needed to evaluate the conclusions of this study are present in the paper and/or the supplementary materials. Source data are provided with this paper.

Human research participants

Policy information about studies involving human research participants and Sex and Gender in Research.

Reporting on sex and gender	N/A
Population characteristics	N/A
Recruitment	N/A
Ethics oversight	N/A

Note that full information on the approval of the study protocol must also be provided in the manuscript.

Field-specific reporting

Please select the one below that is the best fit for your research. If you are not sure, read the appropriate sections before making your selection.

☒ Life sciences ☐ Behavioural & social sciences ☐ Ecological, evolutionary & environmental sciences

For a reference copy of the document with all sections, see nature.com/documents/nr-reporting-summary-flat.pdf

Life sciences study design

All studies must disclose on these points even when the disclosure is negative.

Sample size	Cryo-EM: 161,092 good particles were obtained from 2,408 micrographs, which are sufficient to get a 3.3 Å cryo-EM map for atomic modeling.<br>LC-MS: three to four independent experiments were performed for LC-MS, which are sufficient to get the reproducible results indicated in this study.
Data exclusions	Cryo-EM particles which have poor qualities or are not interested targets were excluded during 2D and 3D classification. This standard procedure has been widely used to obtain high resolution cryo-EM structures of biomacromolecules.
Replication	SDS-PAGE of the salivary gland exact were performed three times with independent samples. LC/MS for salivary gland exact and saliva were repeat three times and four times with independent samples, respectively. All attempts at replication were successful.
Randomization	Since this study didn't involve research on living organisms, no confounding factors requiring randomization were expected. No randomization has been performed.
Blinding	Since this study didn't involve research on living organisms, no confounding factors requiring binding were expected. No blinding has been performed.

Reporting for specific materials, systems and methods

We require information from authors about some types of materials, experimental systems and methods used in many studies. Here, indicate whether each material, system or method listed is relevant to your study. If you are not sure if a list item applies to your research, read the appropriate section before selecting a response.

Materials & experimental systems

n/a	Involved in the study
☒	☐ Antibodies
☒	☐ Eukaryotic cell lines
☒	☐ Palaeontology and archaeology
☐	☒ Animals and other organisms
☒	☐ Clinical data
☒	☐ Dual use research of concern

Methods

n/a	Involved in the study
☒	☐ ChIP-seq
☒	☐ Flow cytometry
☒	☐ MRI-based neuroimaging

Animals and other research organisms

Policy information about studies involving animals; ARRIVE guidelines recommended for reporting animal research, and Sex and Gender in Research

Laboratory animals	Aedes aegypti (Liverpool strain) mosquitoes (5-7 days old)
Wild animals	No wild animals are used in this study
Reporting on sex	Sugar-fed female adult mosquitoes are used in this study for the reason that only the female mosquitoes are involved in blood feeding and related to disease transmission. In addition, the protein SGS1 is expressed in the female mosquito instead of male ones.
Field-collected samples	No field-collected samples were used in this study.
Ethics oversight	All of the mosquito related experiments were performed in the Department of Entomology and Fralin Life Science Institute, Virginia Tech or the Laboratory of Malaria and Vector Research, NIAID, NIH with established protocols. No ethics guidance is required for the research of mosquito.

Note that full information on the approval of the study protocol must also be provided in the manuscript.
