# Supplementary figures and images for: Native structure of mosquito salivary protein uncovers domains relevant to pathogen transmission
Source: Nat Commun. 2023 Feb 17;14:899. doi: 10.1038/s41467-023-36577-y (PMC9935623; doi:10.1038/s41467-023-36577-y)

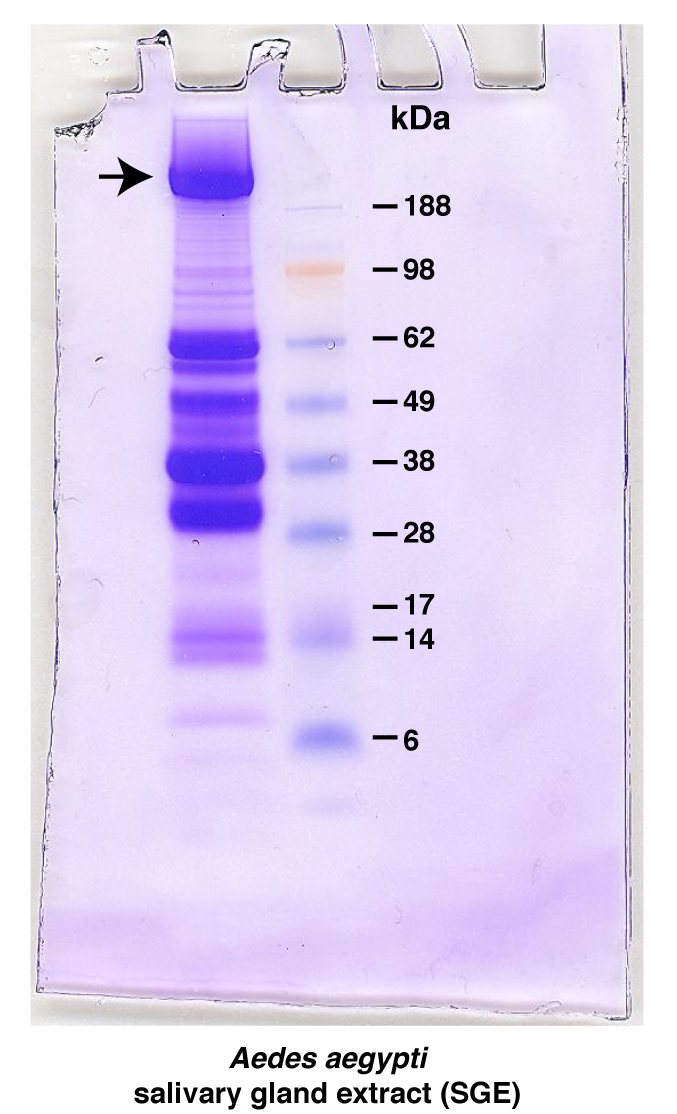

Supplement: Supplementary file 9 — Source Data [file 41467_2023_36577_MOESM9_ESM.zip › uncropped_gel for Supplementary Fig. 1a.tif]
